# Downregulation of lncRNA CDKN2B-AS1 attenuates inflammatory response in mice with allergic rhinitis by regulating miR-98-5p/SOCS1 axis

**DOI:** 10.1007/s10142-024-01318-x

**Published:** 2024-03-04

**Authors:** Bangyu Deng, Yunxia zhao, Jisheng Liu

**Affiliations:** 1 Department of Otolaryngology, First Affiliated Hospital of Soochow University, No.899, Pinghai Road, Suzhou, 215006 Jiangsu China; 2 Department of Otolaryngology–Head and Neck Surgery, Suzhou Affiliated Hospital of Nanjing Medical University, Suzhou, Jiangsu China; 3 Department of Maternal and Child Health, Suzhou Jinji Lake Health Service Center, Suzhou, Jiangsu China

**Keywords:** lncRNA CDKN2B-AS1, Allergic inflammation, miR-98-5p/SOCS1

## Abstract

Long non-coding RNA cyclin-dependent kinase inhibitor 2B antisense RNA 1 (CDKN2B-AS1) in various diseases has been verified. However, the underlying mechanism of CDKN2B-AS1 contributes to the development of allergic rhinitis (AR) remains unknown. To evaluate the impact of CDKN2B-AS1 on AR, BALB/c mice were sensitized by intraperitoneal injection of normal saline containing ovalbumin (OVA) and calmogastrin to establish an AR model. Nasal rubbing and sneezing were documented after the final OVA treatment. The concentrations of IgE, IgG1, and inflammatory elements were quantified using ELISA. Hematoxylin and eosin (H&E) staining and immunofluorescence were used to assess histopathological variations and tryptase expression, respectively. StarBase, TargetScan and luciferase reporter assays were applied to predict and confirm the interactions among CDKN2B-AS1, miR-98-5p, and SOCS1. CDKN2B-AS1, miR-98-5p, and SOCS1 levels were assessed by quantitative real-time PCR (qRT-PCR) or western blotting. Our results revealed that CDKN2B-AS1 was obviously over-expressed in the nasal mucosa of AR patients and AR mice. Down-regulation of CDKN2B-AS1 significantly decreased nasal rubbing and sneezing frequencies, IgE and IgG1 concentrations, and cytokine levels. Furthermore, down-regulation of CDKN2B-AS1 also relieved the pathological changes in the nasal mucosa, and the infiltration of eosinophils and mast cells. Importantly, these results were reversed by the miR-98-5p inhibitor, whereas miR-98-5p directly targeted CDKN2B-AS1, and miR-98-5p negatively regulated SOCS1 level. Our findings demonstrate that down-regulation of CDKN2B-AS1 improves allergic inflammation and symptoms in a murine model of AR through the miR-98-5p/SOCS1 axis, which provides new insights into the latent functions of CDKN2B-AS1 in AR treatment.

## Introduction

Allergic rhinitis (AR), the prevailing inflammatory disorder affecting the nasal mucosa, is primarily driven by serum immunoglobulin E (IgE) upon exposure to allergens and is distinguished by excessive mucus secretion (Helman et al. 2020). Currently, anti-inflammatory drugs, antihistamines, leukotrienes and glucocorticoids are used to treat AR (Nieto et al. 2021; Tojima et al. 2019). Similar to other allergic diseases, the etiology of AR is intricate and indeterminate. Consequently, there is a pressing need to identify the key pathogenic molecules involved in AR pathology and sought for the innovative therapeutic targets for this condition.

LncRNAs are a type of RNA with more than 200 nucleotides but no open reading frames (Kumar and Goyal 2017). LncRNAs can indirectly regulate gene coding by sponging miRNAs, thus affecting downstream gene expression. Cyclin-dependent kinase inhibitor 2B antisense RNA 1 (CDKN2B-AS1) is connected with the development of diabetes, coronary heart disease, atherosclerosis and cancers (Dasgupta et al. 2020). Studies have shown that CDKN2B-AS1 acts as a sponge for miR-98-5p. Previous study indicated that CDKN2B-AS1 regulates E2F2 by sponging miR-98-5p to promote the proliferation, clone formation, and invasion of nasopharyngeal carcinoma (NPC) cells. The CDKN2B-AS1/miR-98-5p axis plays an essential role in the occurrence and progression of NPC, it is may be a treatment target and prognostic marker for patients with NPC (Li et al. 2021). Furthermore, studies have shown that CDKN2B-AS1 regulated NOTCH2 expression by sponging miR-98-5p. CDKN2B-AS1 participates in high glucose-induced apoptosis and fibrosis via NOTCH2 by functioning as a miR-98-5p decoy in human podocytes and renal tubular cells (Xiao et al. 2021). To date, few reports have elucidated the functions of lncRNA CDKN2B-AS1 in AR animal models. Consequently, additional research is warranted to investigate the effects and molecular mechanisms of lncRNA CDKN2B-AS1 on AR.

Thus, based on previous studies, our study was designed to analyze the role of lncRNA CDKN2B-AS1 in AR mice and elucidate its underlying mechanisms in mediating the miR-98-5p/SOCS1 axis. In this study, we hypothesized that (i) lncRNA CDKN2B-AS1 may be a significant regulator for AR progression; and (ii) the latent mechanisms of lncRNA CDKN2B-AS1’s protective effects may be associated with the miR-98-5p/SOCS1 axis. Our findings show that downregulation of the lncRNA CDKN2B-AS1 improves allergic inflammation and symptoms in an AR model by regulating the miR-98-5p/SOCS1 axis, indicating that the lncRNA CDKN2B-AS1 may be a promising agent for AR diagnosis and treatment.

## Materials and methods

### Clinical specimen collection

The inclusion and exclusion criteria were based on previous study (Nasiri Kalmarzi et al. 2019). The inclusion criteria were as follows: diagnosed with AR, within the age range of 18—60 years old, and being able to provide informed consent. The exclusion criteria were as follows: inflammatory diseases, including autoimmune or infectious diseases, as well as receiving corticosteroids or immunotherapy within the past month; patients with other types of rhinitis, such as, infectious, drug-induced, and hormonal rhinitis; a positive history of any allergic diseases other than AR. Based on the inclusion and exclusion criteria, 30 patients with AR were selected from the otorhinolaryngology surgery clinic at the First Affiliated Hospital of Soochow University. The nasal mucosa tissues of AR patients were obtained in the outpatient treatment room and stored in an 80℃ refrigerator. Thirty patients with non-atopic obstructive snoring who underwent adenoidectomy during the same period were selected as controls. None of the patients underwent hormone and antihistamine remedies within 1 month or signed an informed consent form. This research was approved by the Ethics Committee of the First Affiliated Hospital of Soochow University.

### Animals

BALB/c mice, aged 8 weeks, were obtained from Nanfang Model Biotechnology and placed in a dark/light cycle of 12 h at room temperature with free access to food and water. Mice were provided ad libitum access to water and standard rodent chow during the experiment. This study was approved by the Animal Use and Nursing Committee of First Affiliated Hospital of Soochow University.

To establish an AR mice model, mice were sensitized with OVA. On days 0, 7 and 14, 200 μL of normal saline, which contains 25 μg OVA and 2 mg aluminum hydroxide was intraperitoneally injected into mice. One week after the last treatment, 20 μL saline with 3% OVA was used for secondary immunization in mice for one week. All the mice were successfully injected into AR mice by OVA. The control group was administered saline without OVA or aluminum.

### Quantitative real-time PCR (qRT-PCR) analysis

After treatment, total RNAs were collected using the TRIzol reagent (Beyotime, Shanghai, China) according to the manufacturer’s instructions. RNAs were reversely transcribed to cDNAs by using cDNA Synthesis Kit (Thermo Fisher Scientific, USA). The sequences of the primers are listed as follows: forward primer for CDKN2B-AS1: 5’ -GAAGATCTGGAGCAGGAACCAC-3’; reverse primer for CDKN2B-AS1: 5’-GTCAATCAGAGCAAACTGCA GTG-3’; forward primer for miR-98-5p: 5’-CCCGGGTGAGGTAGTAAGTTG-3’; reverse primer for miR-98-5p: 5’-CTCAACTGGTGTCGTGGAGTC-3’; forward primer for U6: 5’-CTCGCTTCGGCAGCACAT-3’; reverse primer for U6 5’-AACGCTTCACGAATTTGCGT-3’; forward primer for SOCS1: 5’-CAACGGAACTGCTTCTTCGC-3’; reverse primer for SOCS1 5’-CTCGAAAAGGCAGTCGAAGG-3’; forward primer for GAPDH: 5’-TGAAGGGTGGAGCCAAAAG-3’; reverse primer for GAPDH 5’-AGTCTTCTGGGTGGCAGTGAT-3’. Reaction conditions: 5 min 95℃ followed by 40 cycles of 15 s 95℃, 15 s 58℃ and 32 s 72℃. qRT-PCR analysis was performed using QuFast SYBR Green PCR Master Mix (ELK Biotechnology, China) on an ABI 7500 Real-Time PCR System. The expression of targeted genes was calculated using 2^−ΔΔCt^ method, in which U6 and GAPDH, whose stability was determined by an online software RefFinder (Xie et al. 2023).

### Symptom score

Allergic symptoms (sneezing and nasal itching) were observed and measured within 10 min of the last OVA attack. Symptom scores were calculated as previously described (Ye et al. 2022). Sneezing and nasal itching were graded from one to three based on the degree of severity. For sneezing, a score of 1 indicated fewer than three sneezes within 10 min, 2 indicated four to ten sneezes within 10 min, and 3 indicated more than 11 sneezes within 10 min. For nasal itching, a score of 1 indicated slight occasional scratching within 10 min of the last challenge, 3 indicated severe and persistent scratching within 10 min of the last challenge, and 2 indicated itching between these frequencies. AR mice that did not achieve a score of more than 4 were excluded from further research.

### ELISA

The concentrations of OVA-specific IgE and IgG1 in serum and the secretion of TNF-α, IL-4, IL-5, and IFN-γ in serum, nasal lavage fluid and lung tissue of mice were assessed by ELISA kits (Beyotime) following the instructions of the manufacturer. The OD_450nm_ was assessed using luminometer (AutoLumat Plus, Germany).

### Histological analysis

Mice were euthanized 24 h after intranasal sensitization. Based on the size of the required section area, fresh mouse tissues were soaked in 5% glacial acetic acid aqueous solution for 10–20 min and frozen. The frozen tissue was cut into flake tissue blocks of approximately 2-mm thickness and embedded in paraffin. The sections were then treated, and the pathological changes in the nasal mucosa were investigated using a Hematoxylin and Eosin Staining Kit (Beyotime). The sections were stained with Sirius red to determine eosinophil infiltration in the nasal mucosa (Ansari and Nikpour 2023). The number of eosinophils in the nasal mucosa was quantified at 400 × magnification.

### Laser scanning confocal microscopy (LSCM) analysis

Tryptase expression in mast cells was investigated using LSCM. Tissue sections were cultivated with monoclonal Rabbit anti mouse tryptase antibody with known markers of hypertrophic cells at 4℃ overnight. The morphology of mast cells, distribution, secretion of tryptase, and number of mast cells in each group were detected and quantified using FITC green fluorescence.

### Dual-luciferase reporter assay

StarBase and TargetScan were used to identify the correlation between miR-98-5p and lncRNA CDKN2B-AS1 or SOCS1. As SOCS1 and miR-98-5p for example, the 3'-UTR of miR-98-5p containing putative or mutated SOCS1 binding site was amplified by PCR and then cloned into a pmirGLO vector (Promega, USA) to form the reporter vector miR-98-5p-wild-type miR-98-5p-WT). Another reporter vector was also obtained by inserting a mutated binding site and was named as SOCS1-mutated-type (SOCS1-MUT). miR-98-5p-WT or miR-98-5p-MUT and SOCS1 mimics or mimic controls were co-transfected into 293 T cells using Lipofectamine 2000 (Invitrogen, USA) following by the manufacturer’s protocol.

### Western blotting

SOCS1 expression was determined using western blotting. Proteins were extracted using RIPA buffer (Beyotime) and quantified using a BCA protein assay kit (ASPEN, AS1086). Equal amounts of samples were separated by 10% SDS-PAGE and transferred to PVDF membrane. After the membrane blocked with 5% non-fat milk for 1 h, the membranes were probed with primary antibodies against SOCS1(1: 1000 dilution), GAPDH (1: 1000 dilution) overnight at 4℃. After washing with TBST, the membranes were incubated with horseradish peroxidase-labelled secondary antibodies (HRP-labeled secondary antibodies, 1:10,000) for 1 h. Specific bands were visualized using Western blotting substrate (Pierce, USA).

### Statistical analysis

Statistical analyses were performed using GraphPad Prism, and all data are presented as mean ± standard deviation (SD). All experiments were performed in triplicate. Differences between the two groups were analyzed using Student's t-test. Differences among groups were analyzed using one-way ANOVA, followed by Tukey’s test. P < 0.05 was considered to be significant difference.

## Results

### LncRNA CDKN2B-AS1 was up-regulated in the nasal mucosa tissues of AR patients and AR mice

**Fig. 1:**
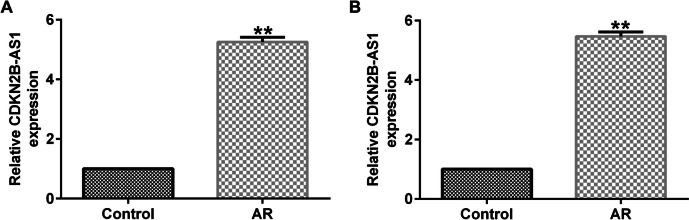
Expression of lncRNA CDKN2B-AS1 in the nasal mucosa tissues of AR patients and mice. (**A**) lncRNA CDKN2B-AS1 expression in AR patients, n = 30; (**B**) lncRNA CDKN2B-AS1 expression in AR mice. n = 3. ^**^P < 0.01 vs. the Control group

First, we detected the levels of lncRNA CDKN2B-AS1 in AR patients and mice. Results from Fig. 1A-B demonstrated that lncRNA CDKN2B-AS1 was remarkably over-expressed in the nasal mucosal tissues (P < 0.01), revealing that lncRNA CDKN2B-AS1 may be a latent mediator in the occurrence and development of AR.

### Suppression effects of LncRNA CDKN2B-AS1 on OVA-induced IgE, IgG1 concentration and allergic response in AR mice

**Fig. 2:**
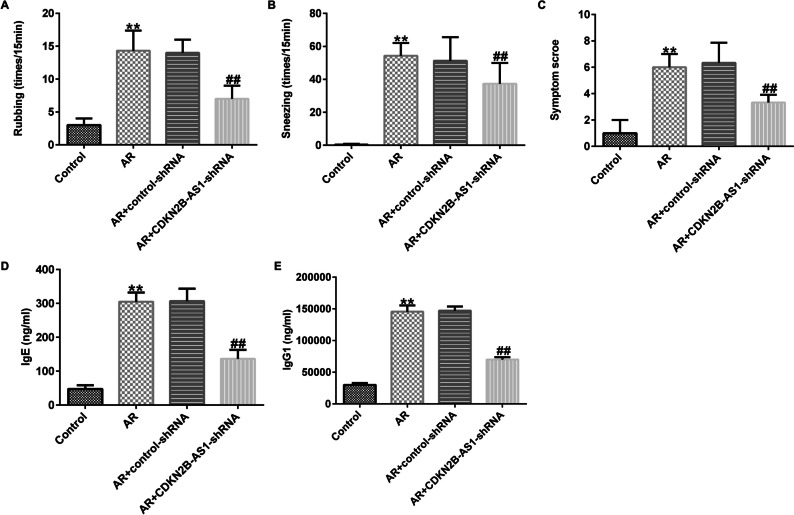
Effects of lncRNA CDKN2B-AS1 on OVA-induced IgE and IgG1 concentration and allergic response in AR mice. (**A**) The rates of rubbing; (**B**) The rates of sneezing; (**C**) Symptom scores. (**D**) OVA-induced IgE concentration were evaluated using ELISA; (**E**) OVA-induced IgG1 concentration were evaluated using ELISA. n = 3. ^**^P < 0.01 vs. Control; ^##^P < 0.01 vs. AR + control-shRNA

To elucidate the role of lncRNA CDKN2B-AS1 in OVA-stimulated allergic symptoms in AR mice, allergic symptoms, including nasal rubbing, sneezing number and symptom score were calculated after the last sensitization on day 34. As displayed in Fig. 2A-C, repeated administration of OVA promoted rubbing and sneezing. Moreover, the symptom score was obviously enhanced in AR mice induced by OVA, the reduced rubbing, sneezing, and symptom scores in lncRNA CDKN2B-AS1-shRNA treated AR mice was observed. Furthermore, we detected OVA-specific IgE and IgG1 concentrations in AR mouse serum. Our results presented that intranasal administration of CDKN2B-AS1-shRNA suppressed OVA-induced IgE and IgG1 expression (Fig. 2D-E, P< 0.01). These results implied that down-regulation of lncRNA CDKN2B-AS1 alleviated OVA-specific IgE, IgG1 and allergic responses in AR mice.

### Suppression effects of lncRNA CDKN2B-AS1 on inflammatory cell infiltration into the nasal mucosa tissues

**Fig. 3:**
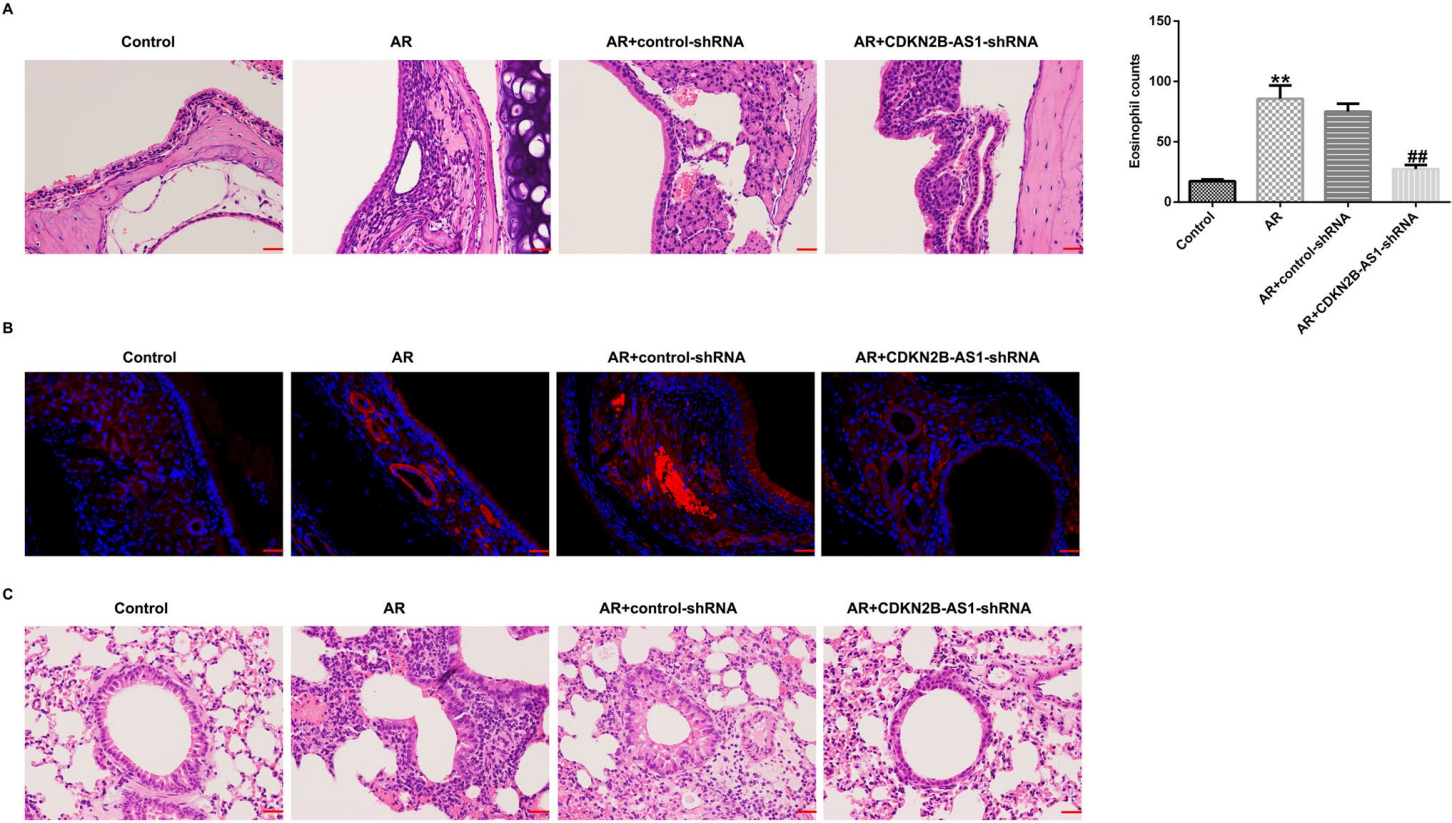
Effects of lncRNA CDKN2B-AS1 on inflammatory cell infiltration in the AR mice nasal mucosa tissues. (**A**) Representative images of pathological modification in nasal mucosa and quantification of eosinophil infiltration numbers in each group (scar bar = 50 μm). (**B**) LSCM was conducted to assess tryptase (red) expression in mast cells (scar bar = 20 μm). (**C**) Representative images of lung tissue structure injury were investigated by H&E staining (scar bar = 50 μm). n = 3. ^**^P < 0.01 vs. Control; ^##^P < 0.01 vs. AR + control-shRNA

We demonstrated the functions of lncRNA CDKN2B-AS1 in pathological changes in AR mice. OVA stimulation led to obviously pathological alterations such as congestion, dropsy, and structural abnormalities of the nasal mucosal epithelium. Moreover, H&E staining revealed that the number of eosinophils in AR mice were significantly higher than those in the Control group (Fig. 3A, P< 0.01). We also observed structural lung tissue injury in AR mice (Fig. 3C). However, these phenomena were relieved after lncRNA CDKN2B-AS1-shRNA treatment. Furthermore, LSCM results revealed an obvious reduction in the number of mast cells in AR mice induced by lncRNA CDKN2B-AS1-shRNA (Fig. 3B). Our findings indicated that lncRNA CDKN2B-AS1-shRNA could relieve pathological alterations and immune cell infiltration in the AR mice nasal mucosa.

### LncRNA CDKN2B-AS1 down-regulation suppressed the secretion of cytokines in AR mice

**Fig. 4:**
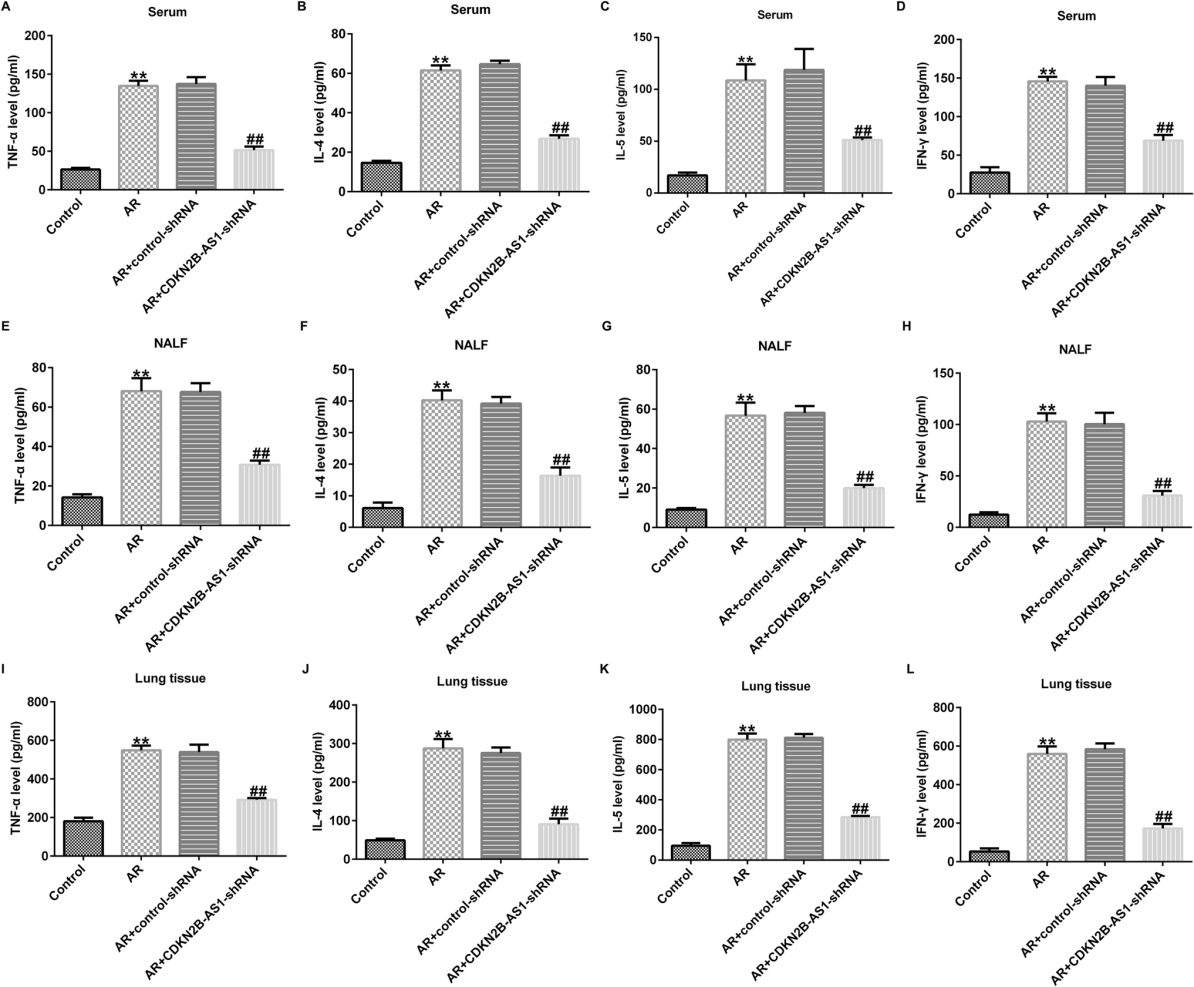
Effects of lncRNA CDKN2B-AS1 on the secretion of cytokines in AR mice. (**A**) TNF-α in serum; (**B**) IL-4 in serum; (**C**) IL-5 in serum; (**D**) IFN-γ in serum. (**E**) TNF-α in nasal lavage fluid; (**F**) IL-4 in nasal lavage fluid; (**G**) IL-5 in nasal lavage fluid; (**H**) IFN-γ in nasal lavage fluid. (**I**) TNF-α in lung tissues; (**J**) IL-4 in lung tissues; (**K**) IL-5 in lung tissues; (**L**) IFN-γ in lung tissues. n = 3. ^**^P < 0.01 vs. Control; ^##^P < 0.01 vs. AR + control-shRNA

We verified the effect of lncRNA CDKN2B-AS1 on pro-inflammatory factors in AR mice by detected TNF-α, IL-4, IL-5 and IFN-γ levels in the serum, nasal lavage fluid, and lung tissues. As shown in Fig. 4, the levels of inflammatory cytokines were higher in the serum, nasal lavage fluid, and lung tissues of AR mice than those mice in the Control group (P < 0.01). Nevertheless, these levels were markedly reduced by lncRNA CDKN2B-AS1-shRNA, suggesting that the down-regulation of lncRNA CDKN2B-AS relieved the inflammatory response in AR mice.

### MiR-98-5p directly interacted with lncRNA CDKN2B-AS1

**Fig. 5:**
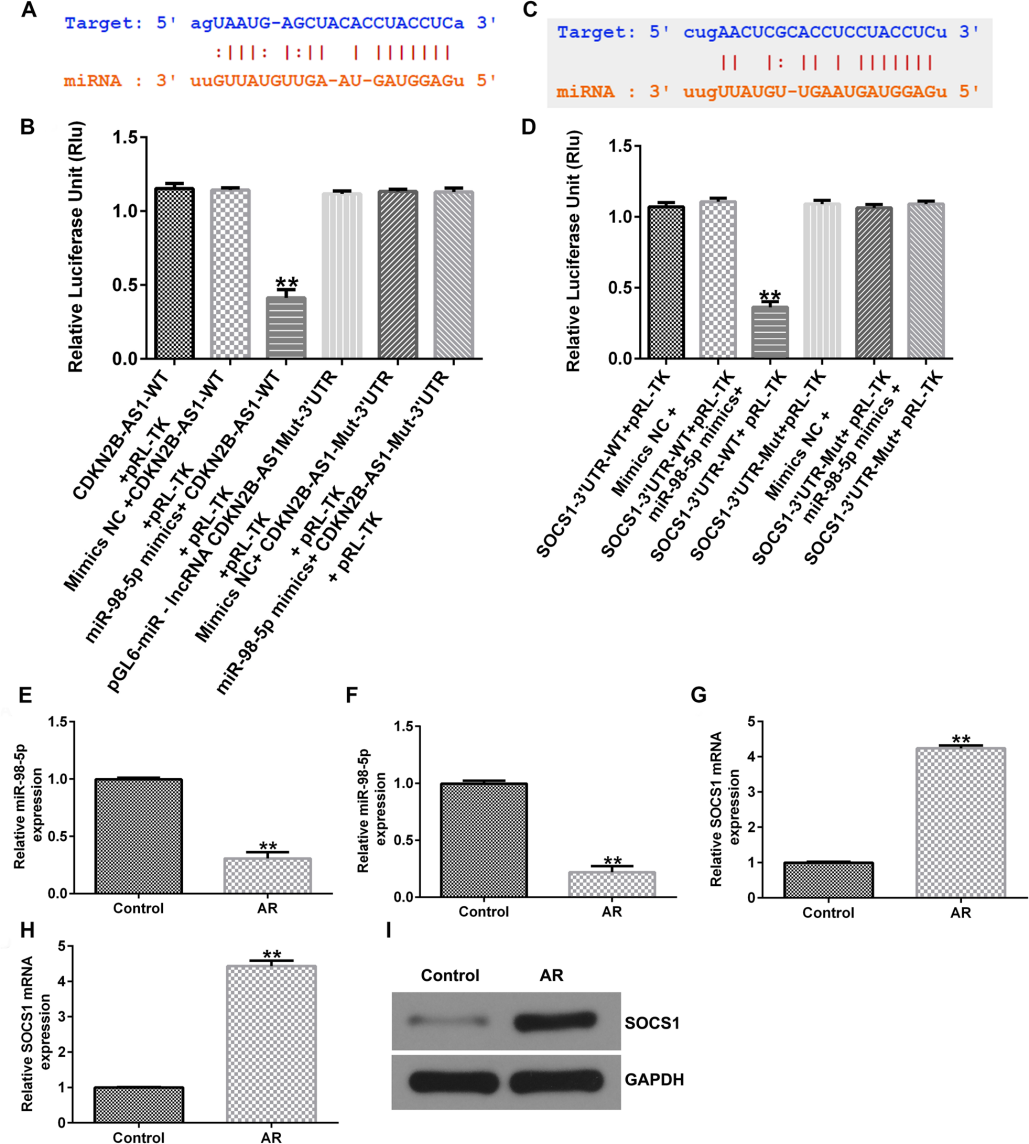
Dual luciferase reporter gene system analyzed the combination of genes. (**A**) The complementary sequences of lncRNA CDKN2B-AS1 and miR-98-5p were displayed. (**B**) Relationship between miR-98-5p and lncRNA CDKN2B-AS1 were verified by dual luciferase reporter gene system assay. (**C**) A schematic diagram of the forecasted lncRNA CDKN2B-AS1 binding site to miR-98-5p. (**D**) Dual luciferase reporter gene system analyzed the combination of miR-98-5p and lncRNA CDKN2B-AS1. (**E**) MiR-98-5p levels in AR patients’ nasal mucosa tissue; (F) MiR-98-5p levels in AR mice nasal mucosa tissue. (**G**) SOCS1 levels in AR patients’ nasal mucosa tissue; (**G**) SOCS1 levels in the nasal mucosa tissue of AR mice; (**I**) Determination of SOCS1 expression using Western blot analysis. n = 3. ^**^P < 0.01 vs. control

To determine whether CDKN2B-AS1 acts as an endogenous competitive RNA against specific miRNAs, we used StarBase to identify candidate sites, and observed that miR-98-5p was a potential target of CDKN2B-AS1 (Fig. 5A). Furthermore, dual-luciferase reporter system confirmed the relationship between lncRNA CDKN2B-AS1 and miR-98-5p, suggesting that the lncRNA CDKN2B-AS1 plasmid substantially reduced the luciferase activity of the miR-98-5p-WT reporter. However, no significant changes were observed in the miR-98-5p-MUT group (Fig. 5B). Our results demonstrated that miR-98-5p directly interacts with lncRNA CDKN2B-AS1.

### SOCS1 directly interacted with miR-98-5p

To better explain the mechanisms of miR-98-5p in AR mice, TargetScan was applied for checking the target sites between miR-98-5p and SOCS1, the predicted results were presented in Fig. 5C. Further dual-luciferase reporter analysis revealed that miR-98-5p mimic memorably reduced the luciferase activity of SOCS1-WT reporter, while no significant effect on SOCS1 3’UTR-mut reporter (Fig. 5D). Taken together, our data demonstrated that SOCS1 directly interacts with miR-98-5p.

### MiR-98-5p was down-expressed and SOCS1 was over-expressed in AR patients and mice nasal mucosa tissue

We also detected the levels of miR-98-5p and SOCS1 in the AR mice. qRT-PCR analysis revealed that miR-98-5p was down-regulated in AR patients and AR mice (Fig. 5E-F, P< 0.01). Moreover, we found that the SOCS1 mRNA level was higher in the nasal mucosa tissue of AR patients than those in control group (Fig. 5G, P< 0.01). In addition, SOCS1 mRNA and protein expression were assessed high expression by qRT-PCR (Fig. 5H) and western blotting (Fig. 5I) in the nasal mucosal tissue of AR mice. In summary, our findings suggest that miR-98-5p is downregulated and SOCS1 is overexpressed in AR patients and mice, which may mediate the progression of AR.

### Downregulation of miR-98-5p reversed the effects of lncRNA CDKN2B-AS1-shRNA on OVA-induced IgE, IgG1 concentration and allergic response in AR mice

**Fig. 6:**
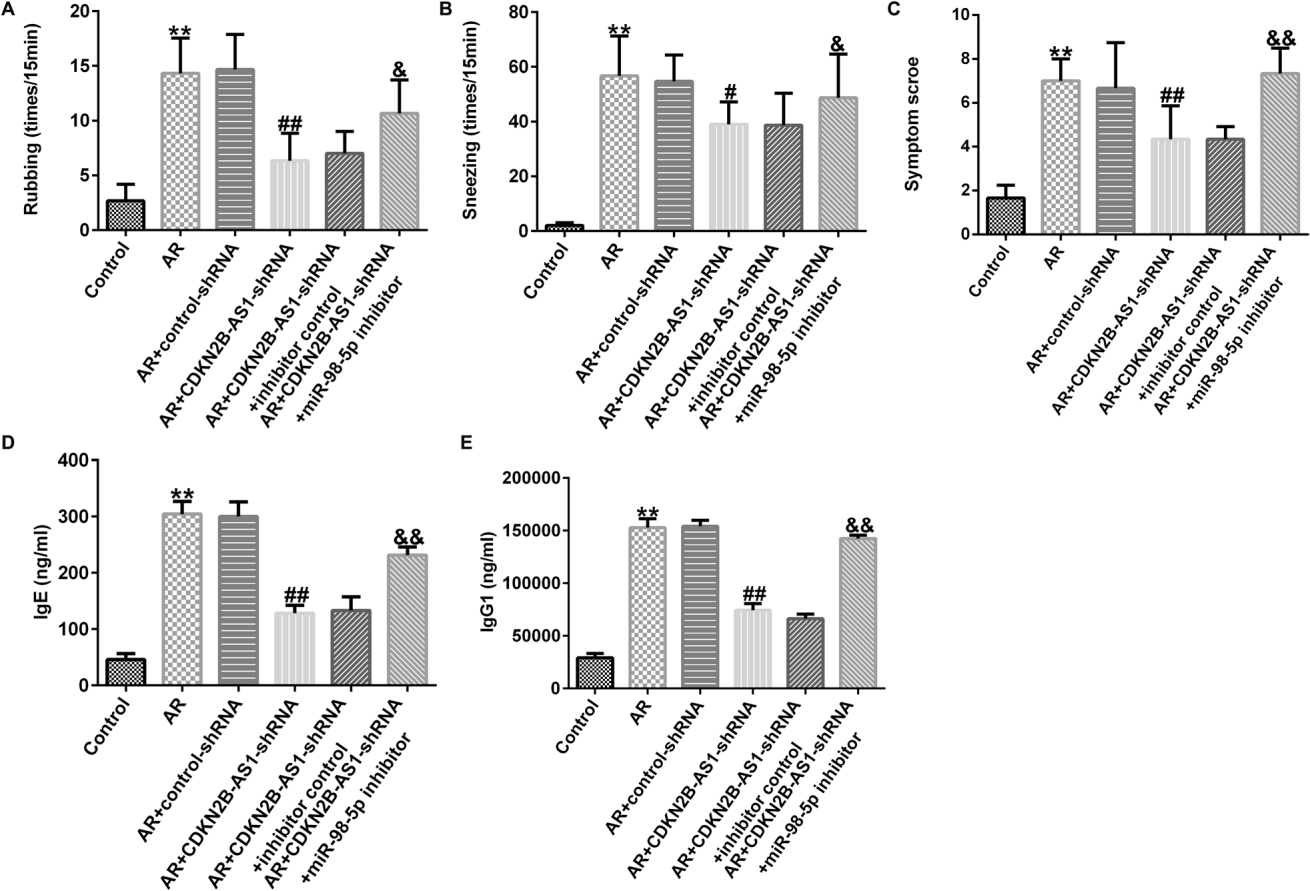
Effects of miR-98-5p suppression on OVA-induced IgE, IgG1 concentration and allergic response in AR mice. The rubbing (**A**) and sneezing (**B**) rates were collected within 15 min of the last OVA induction on day 34. (**C**) Symptom scores were counted. OVA-stimulated IgE (**D**) and IgG1 (**E**) concentrations were analyzed by ELISA. n = 3. ^**^P < 0.01 vs. Control; ^#^P < 0.05, ^##^P < 0.01 vs. AR + control-shRNA; ^&^P < 0.05, ^&&^P < 0.01 vs. AR + CDKN2B-AS1-shRNA + inhibitor control

To explore whether lncRNA CDKN2B-AS1 effects OVA-induced IgE, and IgG1 concentrations and allergic responses by regulating miR-98-5p, AR mice were stimulated with control shRNA, CDKN2B-AS1-shRNA, inhibitor control, or miR-98-5p inhibitor. Results from Fig. 6A-C revealed the inhibition rubbing, sneezing, and symptom scores in the lncRNA CDKN2B-AS1-shRNA treated AR mice. In addition, we observed that intranasal administration of lncRNA CDKN2B-AS1-shRNA reduced OVA-specific IgE and IgG1 concentrations (Fig. 6D-E). Nevertheless, these results were reversed after miR-98-5p inhibitor treatment, indicating that the downregulation of lncRNA CDKN2B-AS1 alleviated the OVA-specific IgE, IgG1, and allergic responses in AR mice by regulating miR-98-5p.

### MiR-98-5p inhibitor reversed the functions of lncRNA CDKN2B-AS1-shRNA in inflammatory cell infiltration in the nasal mucosa tissues

**Fig. 7:**
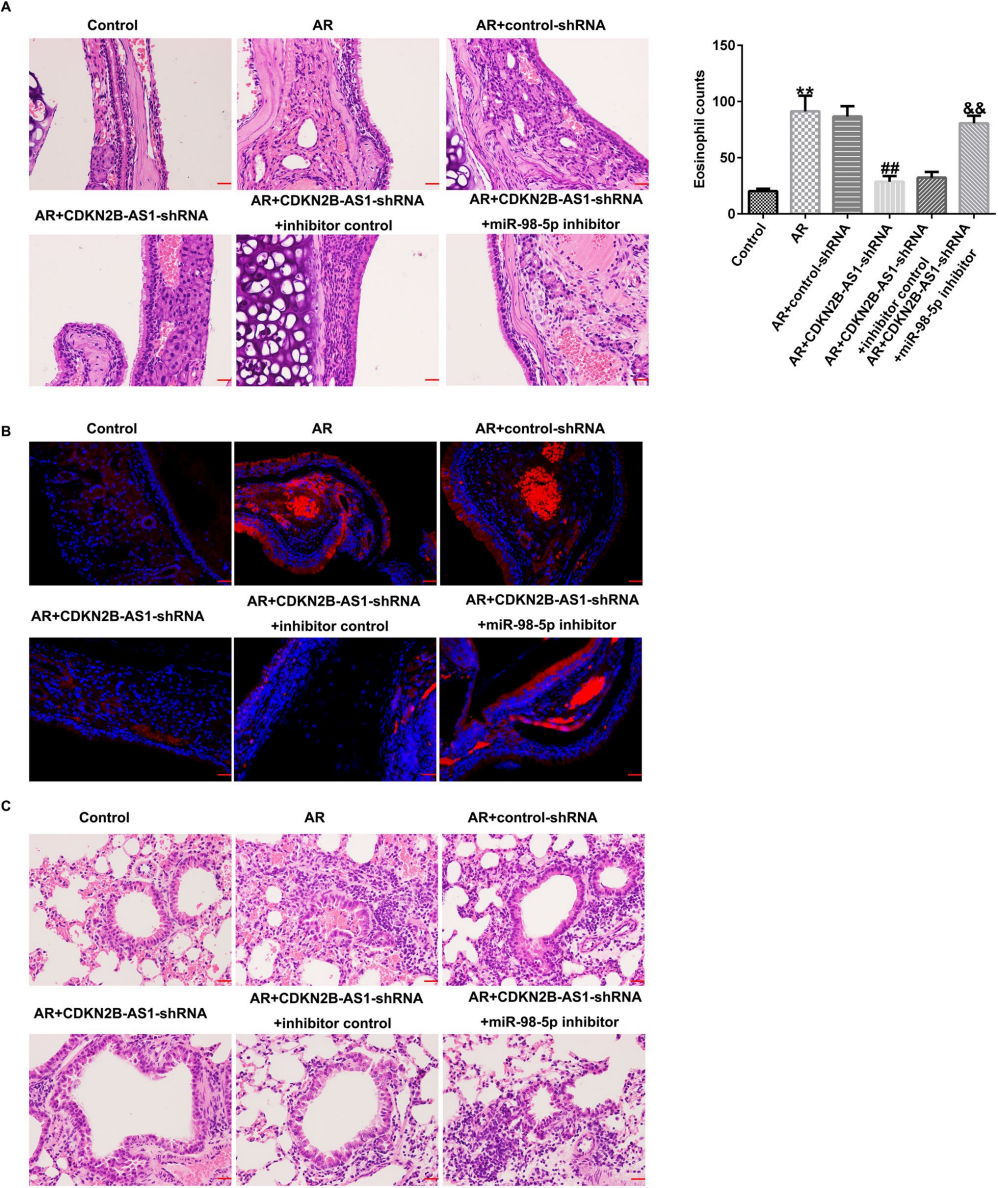
Effects of miR-98-5p inhibitor on inflammatory cell infiltration in AR mice nasal mucosa tissues. (**A**) Representative images of pathological modification in nasal mucosa and the count of eosinophil infiltration were observed (scar bar = 50 μm). (**B**) Tryptase (red) expression in mast cells was visualized by a laser scanning confocal microscopy (scar bar = 20 μm). (**C**) Representative images of lung tissue structure injury were displayed using H&E staining (scar bar = 50 μm). n = 3. ^**^P < 0.01 vs. Control; ^##^P < 0.01 vs. AR + control-shRNA; ^&&^P < 0.01 vs. AR + CDKN2B-AS1-shRNA + inhibitor control

Further, we determined whether lncRNA CDKN2B-AS1 regulates OVA-induced inflammatory cell infiltration into nasal mucosal tissues by regulating miR-98-5p. As illustrated in Fig. 7A-B, lncRNA CDKN2B-AS1-shRNA relieved OVA sensitization-induced pathological alterations, and eosinophil numbers in the nasal mucosa tissue of AR mice were significantly reduced. Moreover, lncRNA CDKN2B-AS1-shRNA alleviated lung tissue structural injury (Fig. 7E). However, these phenomena were reversed by the miR-98-5p inhibitor. In addition, results from LSCM presented an obvious reduction in the number of mast cells in the AR + CDKN2B-AS1-shRNA group (Fig. 7C-D), and this reduction was reversed by the miR-98-5p inhibitor treatment, revealing that the down-regulation of lncRNA CDKN2B-AS1 alleviated the OVA-specific pathological alterations and immune cell infiltration in AR mice by regulating miR-98-5p.

### MiR-98-5p inhibitor reversed the influence of lncRNA CDKN2B-AS1-shRNA on the secretion of cytokines in AR mice

**Fig. 8:**
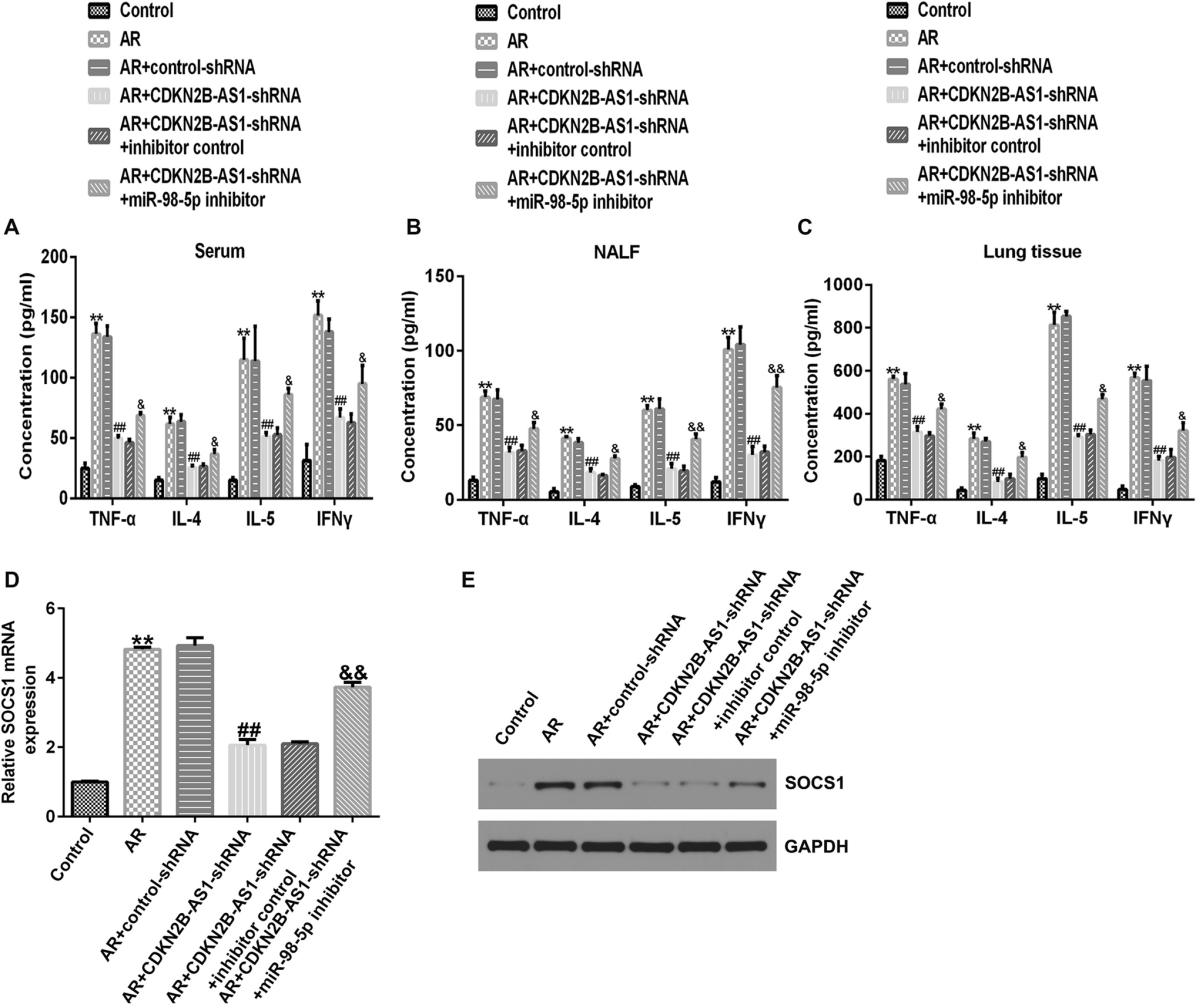
Effects of miR-98-5p inhibitor on the cytokine’s secretion and SOCS1 levels in AR mice. Levels of cytokines (**A**) nasal lavage fluid (**B**) and lung tissues (**C**) in AR mice were determined using ELISA. (**D**) qRT-PCR analysis of SOCS1 levels in each group. (**E**) Determination of SOCS1 level using Western blot assay. n = 3. ^**^P < 0.01 vs. Control; ^#^P < 0.05, ^##^P < 0.01 vs. AR + control-shRNA; ^&^, ^&&^P < 0.05, 0.01 vs.AR + CDKN2B-AS1-shRNA + inhibitor control

We determined the roles of miR-98-5p in the secretion of cytokines, including TNF-α, IL-4, IL-5, and IFN-γ in the serum, nasal lavage fluid, and lung tissues of lncRNA CDKN2B-AS1-shRNA induced AR mice. Results from Fig. 8A-C suggested that inflammatory cytokine levels in the serum, nasal lavage fluid and lung tissues were dramatically inhibited after lncRNA CDKN2B-AS1-shRNA stimulation, and this suppression was reversed by the miR-98-5p inhibitor, indicating that down-regulation of lncRNA CDKN2B-AS relieved the inflammatory response in AR mice by regulating miR-98-5p.

### Down-regulation of miR-98-5p reversed the effects of lncRNA CDKN2B-AS1-shRNA on SOCS1 levels in AR mice nasal mucosa tissues

To further elucidate the latent mechanism of miR-98-5p, we detected the levels of SOCS1 in the nasal mucosal tissues of AR mice. AR mice were treated by control-shRNA, CDKN2B-AS1-shRNA, inhibitor control, or miR-98-5p inhibitor. Results from qRT-PCR and western blot analysis suggested that CDKN2B-AS1-shRNA reduced the level of SOCS1 in the nasal mucosa tissue, whereas this reduction was reversed after the miR-98-5p inhibitor treatment (Fig. 8D-E). Our findings demonstrate that downregulation of CDKN2B-AS1 ameliorates allergic inflammation and symptoms in AR mice by regulating the miR-98-5p/SOCS1 axis.

## Discussion

AR, one of the most common diseases encountered in rhinology clinics, is mediated by the IgE response (Orban et al. 2021). Therefore, it is crucial to develop novel treatments that can effectively block AR development. In the present study, we found that CDKN2B-AS1 and SOCS1 were high expression, whereas miR-98-5p was low expression in the nasal mucosa tissues of patients and mice with AR. In addition, down-regulation of CDKN2B-AS1 significantly decreased nasal rubbing and sneezing frequencies, IgE and IgG1 concentrations, and cytokine levels. Furthermore, the pathological changes in the nasal mucosa, infiltration of eosinophils, and mast cells were relieved. Importantly, all of the above results were reversed by the miR-98-5p inhibitor, whereas miR-98-5p negatively regulated SOCS1 levels.

lncRNAs, which can regulate gene expression at the epigenetic, transcriptional, and post transcriptional levels, are a hot topic in current research (Loganathan and Doss 2023). There is increasing evidence presents that lncRNA CDKN2B-AS1 was related to enhance risk of AR besides increased inflammatory state (Zhou et al. 2016). However, the molecular mechanism underlying CDKN2B-AS1 expression in AR in vivo models has not yet been elucidated. In the present study, we determined the lncRNA CDKN2B-AS1 levels in patients with AR and AR mice model. We found that lncRNA CDKN2B-AS1 was overexpressed in the nasal mucosa tissues of AR mice, demonstrating that lncRNA CDKN2B-AS1 may mediate the anaphylactic properties of AR.

AR is a type of IgE-mediated non-infectious inflammation characterized by Th2 cell polarization (Zhang et al. 2021). Sensitization with allergens and aluminum can stimulate the immune system to produce IgE antibodies, which subsequently lodge in mast cells to form complex IgE-primed mast cells (Skoner 2001). Repeated exposure to allergens can produce more IgE, activate and degranulate tissue-resident mast cells, and release their mediators, thereby stimulating sensory nerves, leading to increased nasal sensations such as itching, rubbing, and sneezing (Wei et al. 2021). As expected, we found the repeated administration of OVA increased rubbing, sneezing, and symptom scores in AR mice, whereas this effect was decreased by lncRNA CDKN2B-AS1-shRNA. Moreover, lncRNA CDKN2B-AS1-shRNA treatment suppressed OVA-induced IgE and IgG1 concentrations, revealing that down-regulation of lncRNA CDKN2B-AS1 alleviated OVA-specific IgE, IgG1, and allergic responses in AR mice.

Mast cells and eosinophils were involved in allergic inflammation via releasing histamine, prostaglandins, and multiple multifunctional factors (Minai-Fleminger and Levi-Schaffer 2009; Puxeddu et al. 2005). We observed that the number of mast cells and eosinophils in the nasal mucosa tissues of AR mice was increased, and the lung tissue structure was damaged. However, the intranasal administration of CDKN2B-AS1-shRNA prominently relieved lung tissue structural injury and suppressed eosinophil and hypertrophic cell infiltration, indicating that the lncRNA CDKN2B-AS1-shRNA relieved pathological alterations and immune cell infiltration in the nasal mucosa tissues of AR mice.

AR is a common type I hypersensitivity reaction. When atopic individuals inhale allergens, antigen presenting cells (APC) ingest and process the allergens and present them to the initial CD4 + T cells, which differentiate into Th2 cells under the stimulation of IL-4. The secreted cytokines (such as TNF-α, IL-5, and IFN-γ) stimulate B-lymphocyte activation and synthesize and release specific IgE, thereby sensitizing the nasal mucosa (Shinmei et al. 2009). TNF-α, a proinflammatory factor produced by hypertrophic cells and eosinophils, is essential for the nosogenesis of AR (Iwasaki et al. 2003; Mohamad et al. 2021). Th2 cytokines, including IL4, IL5, and IFN-γ cells are also related to the pathogenesis of AR (Bayrak Degirmenci et al. 2018; Zhu et al. 2021). In the current study, we found that CDKN2B-AS1-shRNA suppressed the levels of inflammatory factors in AR mice, implying an anti-inflammatory role for CDKN2B-AS1-shRNA in AR.

The lncRNA/miRNA/mRNA axis is fully evidenced as the major regulatory mechanism of lncRNAs in multiple diseases, including AR (Wang et al. 2021). Previous study has demonstrated that CDKN2B-AS1 can act as a ceRNA for miRNAs to mediate cellular processes (Guo et al. 2018). Our findings revealed that lncRNA CDKN2B-AS1 determined miR-98-5p level through binding to the 3’UTR of miR-98-5p. Moreover, SOCS1 directly interacts with miR-98-5p. We determined miR-98-5p and SOCS1 expression in AR patients and AR mice. We found that miR-98-5p was downregulated and SOCS1 was overexpressed in AR patients and AR mice. We then investigated whether lncRNA CDKN2B-AS1 regulates OVA-induced inflammatory cell infiltration into the nasal mucosa tissues by regulating miR-98-5p. The intranasal administration of the miR-98-5p inhibitor reversed the effect of lncRNA CDKN2B-AS1 shRNA, as evidenced by increased rubbing, sneezing, and symptom score, enhanced OVA-specific IgE and IgG1 concentrations, aggravated lung tissue structure injury, increased mast cell number, and increased inflammatory response. In addition, miR-98-5p inhibitor treatment reversed the decrease in SOCS1 levels induced by CDKN2B-AS1-shRNA in AR mice.

In conclusion, our findings demonstrate that downregulation of CDKN2B-AS1 effectively improves AR pathogenesis by regulating the miR-98-5p/SOCS1 axis, elucidates a novel mechanism of AR occurrence and development, and provides promising therapeutic candidate for AR treatment.

## Data Availability

No datasets were generated or analysed during the current study.

## References

[CR1] Ansari S, Nikpour P (2023). LNCAROD promotes the proliferation and migration of gastric cancer: a bioinformatics analysis and experimental validation. Funct Integr Genomics.

[CR2] Bayrak Degirmenci P, Aksun S, Altin Z (2018). Allergic Rhinitis and Its Relationship with IL-10, IL-17, TGF-β, IFN-γ, IL 22, and IL-35. Dis Markers.

[CR3] Dasgupta P, Kulkarni P, Majid S (2020). LncRNA CDKN2B-AS1/miR-141/cyclin D network regulates tumor progression and metastasis of renal cell carcinoma. Cell Death Dis.

[CR4] Guo F, Tang C, Li Y (2018). The interplay of LncRNA ANRIL and miR-181b on the inflammation-relevant coronary artery disease through mediating NF-κB signalling pathway. J Cell Mol Med.

[CR5] Helman SN, Barrow E, Edwards T (2020). The Role of Allergic Rhinitis in Chronic Rhinosinusitis. Immunol Allergy Clin North Am.

[CR6] Iwasaki M, Saito K, Takemura M (2003). TNF-alpha contributes to the development of allergic rhinitis in mice. J Allergy Clin Immunol.

[CR7] Kumar MM, Goyal R (2017). LncRNA as a Therapeutic Target for Angiogenesis. Curr Top Med Chem.

[CR8] Li Z, Cai X, Zou W (2021). CDKN2B-AS1 promotes the proliferation, clone formation, and invasion of nasopharyngeal carcinoma cells by regulating miR-98-5p/E2F2 axis. Am J Transl Res.

[CR9] Loganathan T, Doss CG (2023). Non-coding RNAs in human health and disease: potential function as biomarkers and therapeutic targets. Funct Integr Genomics.

[CR10] Minai-Fleminger Y, Levi-Schaffer F (2009). Mast cells and eosinophils: the two key effector cells in allergic inflammation. Inflamm Res.

[CR11] Mohamad SA, Safwat MA, Elrehany M (2021). A novel nasal co-loaded loratadine and sulpiride nanoemulsion with improved downregulation of TNF-α, TGF-β and IL-1 in rabbit models of ovalbumin-induced allergic rhinitis. Drug Deliv.

[CR12] NasiriKalmarzi R, Fakhimi R, Manouchehri F (2019). The relationship between B7 homologous 1 with interleukin-4, interleukin-17 and interferon gamma in patients with allergic rhinitis. Expert Rev Clin Immunol.

[CR13] Nieto A, Nieto M, Mazón Á (2021). The clinical evidence of second-generation H1-antihistamines in the treatment of allergic rhinitis and urticaria in children over 2 years with a special focus on rupatadine. Expert Opin Pharmacother.

[CR14] Orban NT, Jacobson MR, Nouri-Aria KT (2021). Repetitive nasal allergen challenge in allergic rhinitis: Priming and Th2-type inflammation but no evidence of remodelling. Clin Exp Allergy.

[CR15] Puxeddu I, Ribatti D, Crivellato E (2005). Mast cells and eosinophils: a novel link between inflammation and angiogenesis in allergic diseases. J Allergy Clin Immunol.

[CR16] Shinmei Y, Yano H, Kagawa Y (2009). Effect of Brazilian propolis on sneezing and nasal rubbing in experimental allergic rhinitis of mice. Immunopharmacol Immunotoxicol.

[CR17] Skoner DP (2001). Allergic rhinitis: definition, epidemiology, pathophysiology, detection, and diagnosis. J Allergy Clin Immunol.

[CR18] Tojima I, Matsumoto K, Kikuoka H (2019). Evidence for the induction of Th2 inflammation by group 2 innate lymphoid cells in response to prostaglandin D(2) and cysteinyl leukotrienes in allergic rhinitis. Allergy.

[CR19] Wang T, Cai W, Wu Q (2021). Exosomal lncRNA Nuclear Paraspeckle Assembly Transcript 1 (NEAT1)contributes to the progression of allergic rhinitis via modulating microRNA-511/Nuclear Receptor Subfamily 4 Group A Member 2 (NR4A2) axis. Bioengineered.

[CR20] Wei X, Zhang B, Liang X (2021). Higenamine alleviates allergic rhinitis by activating AKT1 and suppressing the EGFR/JAK2/c-JUN signaling. Phytomedicine: Int J Phytother Phytopharmacol.

[CR21] Xiao M, Bai S, Chen J (2021). CDKN2B-AS1 participates in high glucose-induced apoptosis and fibrosis via NOTCH2 through functioning as a miR-98-5p decoy in human podocytes and renal tubular cells. Diabetol Metab Syndr.

[CR22] Xie F, Wang J, Zhang B (2023). RefFinder: a web-based tool for comprehensively analyzing and identifying reference genes. Funct Integr Genomics.

[CR23] Ye M, Liu H, Li H (2022). Long-Term Exposure to Sulfur Dioxide Before Sensitization Decreased the Production of Specific IgE in HDM-Sensitized Allergic Rhinitis Mice. J Inflamm Res.

[CR24] Zhang Y, Lan F, Zhang L (2021). Advances and highlights in allergic rhinitis. Allergy.

[CR25] Zhou X, Han X, Wittfeldt A (2016). Long non-coding RNA ANRIL regulates inflammatory responses as a novel component of NF-κB pathway. RNA Biol.

[CR26] Zhu Y, Ye F, Fu Y (2021). MicroRNA-155-5p regulates the Th1/Th2 cytokines expression and the apoptosis of group 2 innate lymphoid cells via targeting TP53INP1 in allergic rhinitis. Int Immunopharmacol.

